# Protocol for end-design-free rebooting of terminally redundant *Pseudomonas* phages in clinical isolates of *Pseudomonas aeruginosa*

**DOI:** 10.1016/j.xpro.2025.104012

**Published:** 2025-08-04

**Authors:** Daigo Yokoyama, Nana Kimura, Haruka Yamamoto, Yoshiaki Sakata, Jumpei Fujiki, Hidetomo Iwano

**Affiliations:** 1 Laboratory of Veterinary Biochemistry, Department of Veterinary Medicine, Rakuno Gakuen University, Ebetsu, Hokkaido, Japan

**Keywords:** Cell culture, Microbiology, Molecular Biology, Biotechnology and bioengineering

## Abstract

Synthetic phage platforms are robust microbiology tools with therapeutic potential against antimicrobial-resistant bacteria. Here, we present a protocol for rebooting *Pseudomonas* phages with a terminally redundant, circularly permuted 65 kbp genome. We describe steps for designing PCR primers to generate DNA fragments, reconstituting the complete linear phage genome, performing seamless *in vitro* assembly, and finally, purifying and electroporating the DNA using a *P. aeruginosa* clinical isolate.

## Highlights

• Steps for constructing 65 kbp *Pseudomonas* phage DNA by genome assembly
• Preparation of competent cells for phage rebooting with *P. aeruginosa* clinical isolates
• Phage DNA purification and electroporation-based rebooting of linear phage genomes
• Synthesis of phages with terminally redundant and circularly permuted genomes

## Abstract (editor highlights)

Publisher’s note: Undertaking any experimental protocol requires adherence to local institutional guidelines for laboratory safety and ethics.

## Graphical abstract

**Figure:**
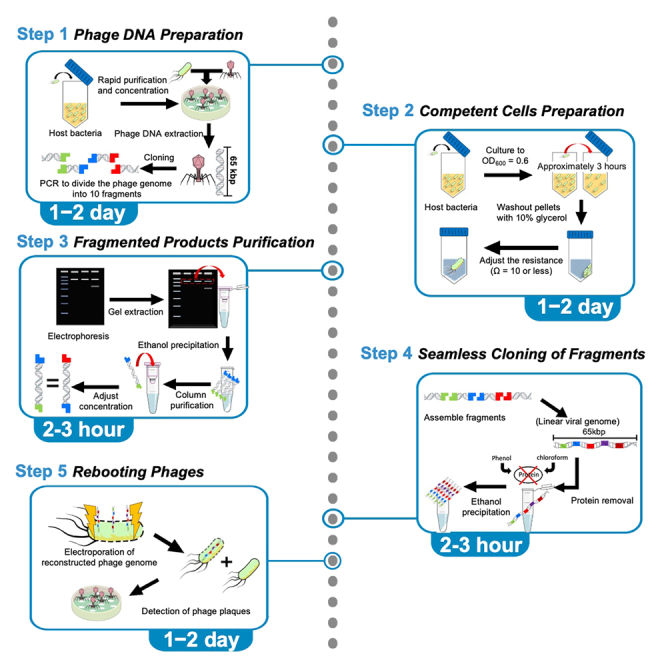

## Before you begin

In recent years, the widespread use of antibiotics in western countries has overshadowed phage therapy.^1^ Nevertheless, with the rapid increase in the proportion of antimicrobial-resistant bacteria, phage therapy has once again gained attention as a powerful therapeutic option.^2^^,^^3^ Moreover, synthetic phage platforms are providing critical insights that drive innovation and advancement in phage therapy.^4^^,^^5^^,^^6^ However, major challenges in rebooting synthetic phages are genome size and defense systems against phages,^7^^,^^8^ because these factors make the process more technically inefficient, less convenient and inhibit phages proliferation. This highlights the need for a straightforward, benchtop protocol for rebooting synthetic phages, given the rise of synthetic phage engineering. In this context, we established an optimized, rapid, and efficient rebooting protocol without cell-free systems^9^^,^^10^ or L-form bacteria^11^^,^^12^ and a phage defense deficient strains,^8^ using a seamless cloning approach^13^ for assembling the complete terminally redundant 65 kbp genome of *Pseudomonas* phage ΦR26 from 10 DNA fragments.

ΦR26 is a member of the *Pbunavirus* genus which has been estimated as circularly permuted genomes with terminal redundancy.^14^^,^^15^^,^^16^ In phages with this genomic structure, the packaging mechanism does not require fixed genome termini, suggesting that it allow us to design of DNA fragments flexibly—assembly can begin from virtually any point in the genome without disrupting functional elements. This property significantly simplifies genome reconstruction compared to phages with direct terminal repeats (DTR) or cohesive ends, where fragment design must preserve exact end structures. Consistent with these reports, we also estimated the termini of ΦR26 using PhageTermVirome^17^ and confirmed that it exhibits terminal redundancy (Figure S1). Importantly, the complete genome of ΦR26 was assembled and successfully rebooted using linear DNA fragments without requiring any special design of viral genomic termini. Our results demonstrate that terminally redundant, circularly permuted phages like ΦR26 are particularly suited for seamless assembly and rebooting, offering a robust platform for synthetic phage applications.

### Innovation

Synthetic phage engineering requires robust protocols for genome reconstruction. This protocol introduces a major innovation by enabling end-design-free assembly and benchtop rebooting of synthetic *Pseudomonas* phages without relying on phage-defense-deficient hosts,^8^ cell-free systems,^9^^,^^10^ or L-form bacteria.^11^^,^^12^ Unlike phages with defined termini such as DTRs or cohesive ends, circularly permuted phages (e.g., *Pseudomonas* virus ΦR26) allow for flexible genome fragmentation and seamless assembly, as any internal site can serve as a junction without disrupting essential genes. This flexibility simplifies genome reconstruction and enables efficient rebooting using a clinical *P. aeruginosa* isolate, thereby expanding the applicability of phage rebooting and accelerating synthetic phage biology and phage therapy.

### Institutional permissions

Experiments using synthetic bacteriophages and *Pseudomonas aeruginosa* strains were conducted under Biosafety Level 2 (BSL-2) conditions in the Rakuno Gakuen University BSL-2 facility in accordance with the Rakuno Gakuen University Biosafety Guidelines.

## Key resources table

**Table:** 

REAGENT or RESOURCE	SOURCE	IDENTIFIER
**Bacterial and virus strains**		

*P. aeruginosa* Pa12	Laboratory Resource Library^3^^,^^18^	GenBank: NZ_CP136598.1
*Pseudomonas* phage R26 (ΦR26)	Laboratory Resource Library^19^	GenBank: NC_048663

**Chemicals, peptides, and recombinant proteins**		

Proteinase K	Takara Bio	Cat#9034
MgSO_4_·7H_2_O	Nacalai tesque	Cat#:21003-75
NaCl	Nacalai tesque	Cat#31320-05
Gelatin	Sigma-Aldrich	Cat#G1890
Tris-HCl pH 7.4	Nippon Gene	Cat#313-90415
Tryptone	Nacalai tesque	Cat#32756-85
Yeast extract	Nacalai tesque	Cat#15838-45
Agarose S	Nippon Gene	Cat#312-01193
BD Bacto Agar	Becton, Dickinson	Cat#63-6531-35
SeaKem ME Agarose	Lonza	Cat#50011
PrimeSTAR Max	Takara Bio	Cat#R045A
Novel Juice Supplied in 6× Loading Buffer	GeneDireX	Cat#LD001-1000
One Mark DNA electrophoresis marker	GeneDireX	Cat#DMF13-0100
1-kb DNA ladder RTU	GeneDireX	Cat#SD010-R500
Etachinmate	Nippon Gene	Cat#312-01791
99.5% Ethanol	Wako Pure Chemical	Cat#057-00451
Phenol:Chloroform:Isoamyl alcohol 25:24:1 mixed, pH 7.9	Nacalai tesque	Cat#25970
Chloroform:Isoamyl alcohol 24:1	Sigma-Aldrich	Cat#C0549
Ethidium bromide (EtBr) solution	Nippon Gene	Cat#315-90051
1 M Tris-HCl (pH 8.0)	Nippon Gene	Cat#314-90065
QuantiFluor dsDNA System	Promega	Cat#E2670

**Critical commercial assays**		

QIAquick PCR Purification Kit	QIAGEN	Cat#28106
QIAquick Gel Extraction Kit	QIAGEN	Cat#28704
Phage DNA Isolation Kit	NorgenBiotek	Cat#46800
TURBO DNA-free Kit	Thermo Fisher Scientific	Cat#AM1907

**Oligonucleotides**		

R26_01_Fw 5′-TTCCCTGGCCGCAACATC-3′	This paper	N/A
R26_01_Rv 5′-CGGTGCTTCCACGCC G-3′	This paper	N/A
R26_02_Fw 5′-TGGACTCACGACGCAATTGC-3′	This paper	N/A
R26_02_Rv 5′-TGGAGCTGTTGCCGAGCG-3′	This paper	N/A
R26_03_Fw 5′-TCGGCATCGATACCGACAAC-3′	This paper	N/A
R26_03_Rv 5′-CAGCTCGTCGAGAAAGACG-3′	This paper	N/A
R26_04_Fw 5′-CCGCAGTTTGTCGAGATTTC-3′	This paper	N/A
R26_04_Rv 5′-GAAAATTTCTCTGATCAGTTACACC-3′	This paper	N/A
R26_05_Fw 5′-GGGTTGATTTAGTGCCCAGG-3′	This paper	N/A
R26_05_Rv 5′-AGCATACTCGTCCTGCGC-3′	This paper	N/A
R26_06_Fw 5′-ATCATGACCAGCCTCGACG-3′	This paper	N/A
R26_06_Rv 5′-CGGTCAGTCCGACCGATC-3′	This paper	N/A
R26_07_Fw 5′-CATGTTCAGATGGACCGTTTC-3′	This paper	N/A
R26_07_Rv 5′-GGCCTGACGGAAAGGTTC-3′	This paper	N/A
R26_08_Fw 5′-TCCAAAATGCAGTTCCGGAGAA-3′	This paper	N/A
R26_08_Rv 5′-ATCGGACAGGTTCGTATCGA-3′	This paper	N/A
R26_09_Fw 5′-GGCGTGAAAGTGATTGGCAT-3′	This paper	N/A
R26_09_Rv 5′-CTAGTGATGAAACCGCCTTG-3′	This paper	N/A
R26_10_Fw 5′-TCAGGGCGCGGCCGG T-3′	This paper	N/A
R26_10_Rv 5′-CTGATAGTACATGTAGTTCTGCG-3′	This paper	N/A

**Other**		

Petri dish with vents, 94 × 16 mm, sterile	Greiner	Cat#633181
Tube, 50 mL, PP, Screw Cap	Greiner	Cat#227261
QSP Microtube 1.5 mL clear 39.5 mm	Thermo Fisher Scientific	Cat#62-7023-67
DISMIC 25CS045AS 39122140	ADVANTEC	CAT#63-1237-36
Centrifugal filter unit (15 mL/100000NMWL)	Merck Millipore	Cat#2-7112-29
Sapphire filter pipette tips, 200 μL	Greiner	Cat#775363
Filter pipette tips 10 μL	NIPPON Genetics	Cat#FGF-10SLA
Filter pipette tips 1,000 μL	NIPPON Genetics	Cat#FGF-10002
0.2 mL 8 strips PCR tube and DomeCap	NIPPON Genetics	Cat#FG-028DC
NEPA cuvette electrode set 2 mm gap 40∼350 μL	NEPA gene	Cat#EC-002S
Disposable bacteria spreader	Kenis	Cat#1-318-0059
Gel maker set-HR [heat-resistant type]	Takara Bio	Cat#GM-HR
BioSpec-nano	Shimadzu	Cat#206−26300−31
Micro-cooled centrifuge	Kubota	Cat#3700
High-speed, large-capacity cooling centrifuge	Kubota	Cat#7000
TOYOBO Fas-III UV transilluminator	Toyobo	Cat#9372959
C1000 Thermal Cycler	Bio-Rad	Cat#1851138
BLooK LED Trans illuminator	GeneDireX	Cat#BK001
Quantus Fluorometer	Promega	Cat#E6150

## Materials and equipment

### Preparation of media and reagents

**Table:** Sodium magnesium (SM) buffer: The following reagents are weighed, and ddH_2_O is added to dissolve the reagents, followed by autoclaving to sterilize (121°C for 20 min)

Reagent	Final concentration	Volume
MgSO_4_·7H_2_O	2.0 g/L	2.0 g
NaCl	5.8 g/L	5.8 g
Gelatin	0.1 g/L	0.1 g
1 M Tris-HCl pH 8.0	50 mM	50 mL
ddH_2_O	N/A	950 mL
Total		1,000 mL

Store at 20°C–25°C for up to 1 year.

**Table:** Luria–Bertani (LB) broth: The following reagents are weighed, and ddH_2_O is added to dissolve the reagents, followed by autoclaving to sterilize (121°C for 20 min)

Reagent	Final concentration	Volume
Tryptone	10 g/L	10 g
NaCl	10 g/L	10 g
Yeast extract	5 g/L	5 g
ddH_2_O	N/A	1,000 mL
Total		1,000 mL

Store at 20°C–25°C for up to 2 months.

**Table:** 50× Tris-acetate-EDTA (TAE) buffer

Reagent	Final concentration	Volume
Tris base	2 M	242 g
Acetic acid	1 M	57.1 mL
EDTA disodium salt dihydrate	50 mM	18.6 g
ddH_2_O	N/A	1,000 mL
Total		1,000 mL

Store at 20°C–25°C for up to 1 year.

**Table:** LB top agar: The following reagents are weighed, and ddH_2_O is added to dissolve the reagents, followed by autoclaving to sterilize (121°C for 20 min)

Reagent	Final concentration	Volume
Tryptone	10 g/L	10 g
NaCl	10 g/L	10 g
Yeast extract	5 g/L	5 g
Agarose ME	5 g/L	5 g
ddH_2_O	N/A	1,000 mL
Total		1,000 mL

Note: Store at 50°C for up to 2 months.

**Table:** LB agar plate: The following reagents are weighed, and ddH_2_O is added to dissolve the reagents, followed by autoclaving to sterilize (121°C for 20 min)

Reagent	Final concentration	Volume
Tryptone	10 g/L	10 g
NaCl	10 g/L	10 g
Yeast extract	5 g/L	5 g
Agar	15 g/L	15 g
ddH_2_O	N/A	1,000 mL
Total		1,000 mL

Note: Store at 4°C for up to 2 months.

***Note:*** Pour the prepared solution into a flat Petri dish and allow it to stand at room temperature until it solidifies.

***Note:*** If water droplets are present on the flat culture medium after cold storage, dry it thoroughly before use.

## Step-by-step method details

### Phage stock preparation

**Timing: 2 days**

The purpose of this section is to prepare a phage stock by rapid purification for the extraction of phage DNA in the following sections.

1. Incubate host bacteria overnight in LB broth at 37°C.
2. Dilute the phage stock to the desired dilution rate in SM buffer.

***Note:*** Diluted phages (approximately 10^6^ PFU/mL) can be used.

3. Warm LB agar (3 plates) in an incubator.
4. Prepare 3 mL of LB top agar in each tube and keep warm at 50°C.
5. Mix 110 μL of host bacteria cultured overnight and 110 μL of phage diluent in a 1.5-mL Eppendorf tube.
6. Add 200 μL of mixture (prepared in Step 5) to 3 mL of LB top agar placed at 50°C.
7. As a negative control, mix 100 μL of host bacteria with 3 mL of LB top agar.
8. Before the prepared LB top agar solidifies, quickly pour it over the LB agar and tilt the plate to distribute it evenly. Let the plate sit undisturbed until the agar solidifies completely.
9. Once the agar has solidified, invert the plate and incubate it overnight at 37°C.

***Note:*** The solidification time is approximately 5 min, and if the agar is not fully solidified, the LB top agar may shift or detach from the plate surface.

10. The next day, check the LB agar plates and pick up those that are lysed in a lacy manner.
11. Add 3 mL of SM buffer to each of the three LB agar plates showing lace-like lysis and incubate them at 37°C for at least 30 min.
12. Collect the lysate solution from the LB agar medium into a 50-mL centrifuge tube by gently scraping it off the top agar using a disposable bacteria spreader.
13. Centrifuge the top agar mixed with the lysate solution at 6,000 *× g* for 10 min.
14. Filter-sterilize the supernatant lysate solution using a 0.45-μm syringe filter unit (Advantec, Tokyo, Japan).
15. Carefully pour the supernatant lysate into a centrifugal filter unit (Merck Millipore, Burlington, MA) and add 10 mL of SM buffer.
16. Securely close the lid and place the centrifuge tube in the centrifuge with the filter face oriented outward, and centrifuge it at 4,000 *× g* for 10 min.
17. Check the filtration level of the lysate solution and centrifuge until 1 mL remains in the upper chamber. Repeat the centrifugation briefly, ensuring that the lysate solution does not completely drain into the lower layer.
18. Repeat the same procedure three times, substituting with SM buffer.

***Note:*** Ensure that the lysate in the upper chamber does not completely drain into the lower chamber.

***Note:*** Because the filtration speed varies based on the type and titer of the phage, it is important to frequently monitor the progression of filtration by performing short centrifugation intervals.

19. When only 1 mL of lysate remains in the upper chamber, discard the lower layer and carefully collect 1 mL of the phage solution in the upper chamber into a 1.5-mL Eppendorf tube simultaneously washing the inner filter section with the remaining phage solution in the upper chamber.
20. Store the collected phage solution at 4°C.

***Note:*** The collected phage solution will maintain titers for approximately 1 month, depending on the type of phage.

***Note:*** Phage samples stored for more than 1 month may have decreased titers; hence, it is recommended to check the titers again.

### Plaque assay for phage titer determination

**Timing: 2 days**

This section aims to determine the current titer of the phage sample. In the rebooting of ΦR26, accurate evaluation of the phage titer was essential for obtaining a sufficient concentration of phage DNA using an appropriate method. Although this protocol was optimized for ΦR26, the approach can be effectively applied to other phages as well, making it a fundamental step in the phage rebooting process.

21. Incubate host bacteria overnight at 37°C in LB broth.
22. Dilute the phage stock to the desired dilution rate in SM buffer.

***Note:*** If you have no idea of the phage titer, it is recommended to prepare 10 dilutions ranging from 10^2^ to 10^10^.

23. Warm three sheets of LB agar plates at 37°C.
24. Heat autoclaved LB top agar in a heat block at 50°C to prevent gelation.
25. Mix 110 μL of host bacteria cultured overnight and 110 μL of phage diluent in a 1.5-mL Eppendorf tube.
26. Add a 200 μL of mixture (prepared in Step 25) to 3 mL of LB Top Agar.
27. As a negative control, mix 100 μL of host bacteria with 3 mL of LB top agar.
28. Before the prepared LB top agar solidifies, quickly pour it over the LB agar and tilt the plate to distribute it evenly. Let the plate sit undisturbed until the agar solidifies completely.
29. Once the agar has solidified (approximately 5 min), invert the plate and incubate it overnight at 37°C.
30. Count the phage plaques and measure the phage titer represented as plaque-forming units per milliliter (PFU/mL).

***Note:*** Bubbles formed when LB top agar is layered over LB agar may be mistaken for plaques. To avoid this, mark or remove them by bursting them with a micropipette tip or other suitable method.

### Extraction of phage DNA

**Timing: 6 h**

This section describes the method for extracting the phage genome from the obtained phage stock. In the rebooting of ΦR26, obtaining high-purity, mutation-free DNA at a sufficient yield was crucial for successful genome reconstruction. Although this protocol was optimized for ΦR26, it can also be applied to other phages. Ensuring the integrity and purity of the extracted DNA is a fundamental requirement for phage rebooting, making this step a critical component of the overall process.

31. Prepare 900 μL of sample adjusted to 10^10^ PFU/ml of the phage solution obtained in the previous section. Use SM buffer for phage dilution.
32. Digest exogenous DNA such as bacterial genomes in the sample using the TURBO DNA-free kit (Thermo Fisher Scientific) according to the manufacturer’s protocol.
33. Add 20 μL of Turbo DNase and 100 μL of DNase buffer to the sample and incubate it at 37°C for at least 30 min.
34. Add 4 μL of DNase inactivation buffer and incubate the sample at room temperature with 2–3 times of inverted mixing occasionally for 5 min.
35. Centrifuge the sample at 10,000 *× g* for 2 min and collect the supernatant except for the precipitated DNase inactivation buffer.
36. Extract the phage DNA using the phage DNA isolation kit (Norgen, Thorold, ON, Canada) according to the manufacturer’s protocol.
37. Determine the DNA concentration of the phage genome sample using Biospec-Nano (Shimadzu, Kyoto, Japan).
38. Dilute the phage genome sample with nuclease-free water to 100 ng/μL.
39. Store the phage genome sample at −20°C.

**Pause point:** For long-term storage, we recommend storing the sample at −80°C.

***Note:*** Repeated freezing and thawing can damage the phage DNA. To minimize this risk, store the samples in small aliquots to reduce the frequency of freezing and thawing.

### Designing DNA fragments

**Timing: 1–2 h**

This section describes the design of DNA fragments for seamless cloning. Specifically, we describe the approach of dividing the genome into 10 fragments for PCR amplification, as applied to the *Pseudomonas* virus ΦR26. Although this method was optimized for ΦR26, the same design principles can be applied to other phages, enabling seamless genome assembly across different phages.

***Note:*** If the target phage is registered in GenBank or a similar database, its sequence can be obtained and used from the National Center for Biotechnology Information database.

***Note:*** In phages with terminal redundancy, it is possible to freely design the starting point for genome assembly. However, care must be taken to avoid disrupting the integrity of coding sequences (CDSs). Fragment boundaries should be placed outside of annotated CDS regions to ensure functional gene preservation.

**Figure 1:**
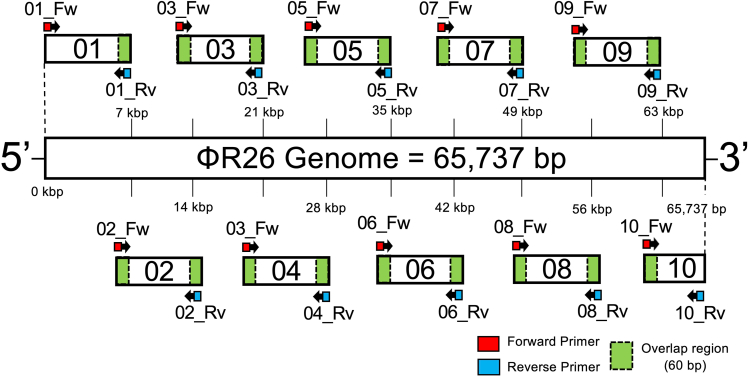
Primer design for amplifying PCR fragments from the ΦR26 genome Primers for ΦR26 genome were designed to provide a 60-bp overlap (green blocks) between each fragment. For the ΦR26 genome, which consists of 65,737 bp, primers were designed to divide the entire genome into 10 fragments. Fragments 1–9 have an approximate product size of 7 kbp, whereas fragment 10 is designed to be 2,737 bp. Arrows with red blocks indicate the forward primers used to amplify each fragment, and arrows with blue blocks indicate reverse primers.
43. For ΦR26 of 65,737 bp, there should be 9 fragments of approximately 7 kbp and 1 fragment of 2,737 bp.

***Note:*** In this case, only the final fragment (fragment 10) will be short; however, this is not a problem because the concentration can be adjusted during seamless cloning.

***Note:*** No overlapping sequences are required on the 5′ side of fragment 1 and 3′ side of fragment 10.

***Note:*** Each primer should be approximately 20 bases long, and the Tm values of the primer sets that amplify the same fragment should be set as close to the same level as possible.

***Note:*** One successful genome assembly was initiated at the CDS for a structural protein (Figure 1), while an alternative construct beginning with the CDS for a non-structural protein (DNA polymerase) was also successfully rebooted in our additional validation.

40. Divide the entire phage sequence into some fragments to be designed (ends design-free), and set up an overlap region of 60 bp.
41. To ensure efficiency in the subsequent seamless cloning, adjust the length per fragment to approximately 7 kbp.
42. Design primer pairs for amplifying each fragment of the phage genome (Figure 1).

### Cloning and purification of phage DNA fragments

**Timing: 1 day**

This section describes the cloning and purification of phage DNA fragments. In the rebooting of ΦR26, obtaining highly purified phage genome fragments with sufficient yield was the most critical factor. Therefore, this step played a vital role in the successful reconstruction of ΦR26 genome. Although this protocol was optimized for ΦR26, it can be broadly applied to other phages as well. Considering its central importance in ensuring the quality and efficiency of phage genome assembly, this section serves as a vital component in the overall phage rebooting process.

44. Set up the PCR system as follows:

**Table:** PCR reaction master mix

Reagent	Amount
Template DNA (10 ng/μL)	1–2 μL
Prime Star Max Ver.2	25 μL
Forward primer (2 μM)	3 μL
Reverse primer (2 μM)	3 μL
ddH_2_O	Up to 50 μL
Total	50 μL

**Table:** PCR cycling conditions

Steps	Temperature	Time	Cycles
Initial Denaturation	98°C	120 s	1 Cycle
Denaturation	98°C	10 s	35 Cycle
Annealing	65°C	45 s	
Extension	72°C	120 s	
Final extension	72°C	120 s	1 Cycle
Hold	4°C	Forever

***Note:*** As the required annealing temperature varies depending on the designed primers, it is strongly recommended to precisely determine the optimal temperature using gradient PCR.

***Note:*** If the yield of DNA is insufficient, it may be improved by extending the extension reaction time.

45. Mix 1 g of agarose S (Nippon Gene, Tokyo, Japan) with TAE buffer. Heat the solution to completely dissolve agarose S in the TAE buffer, and prepare an agarose gel.
46. Pour TAE buffer with agarose S into the electrophoresis bath so that the top of the agarose gel is soaked approximately 1 mm.
47. Mix 10 μL of the DNA fragment sample with 2 μL of 6× loading buffer (Takara Bio, Shiga, Japan).
48. Apply 1-kb DNA ladder RTU (GeneDireX, Taoyuan, Taiwan) to the wells of the gel.
49. Set the electrophoresis conditions to 100 V for 35 min.

***Note:*** If the temperature of the electrophoresis bath exceeds 40°C, the electrophoresis image may become unclear. To prevent this, replace the TAE buffer in the bath with fresh buffer if the bath overheats during the process.

***Note:*** If the gel used for electrophoresis is too thick, the electrophoresis image may become unstable, and the background may become overly bright after ethidium bromide staining, thereby making it difficult to observe the bands.

50. After the completion of electrophoresis, carefully remove the gel and immerse it in ethidium bromide solution.
51. Stain the gel in ethidium bromide solution for 15 min.

**CRITICAL:** Because EtBr is photosensitive, cover the container with aluminum foil.

**Figure 2:**
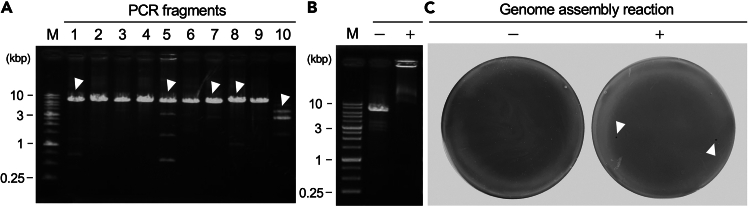
Seamless cloning and rebooting of the ΦR26 genome (A) Electrophoretic analysis of ΦR26 DNA fragments amplified by PCR. Fragments 1–9 exhibited lengths of approximately 7,000 bp each, whereas fragment 10 resulted in approximately 2,700 bp as expected. White arrow heads indicate the bands that should be extracted. (B) Electrophoretic profile of the genome assembly product. (C) After electroporation, the assembled genome was introduced into the P. aeruginosa Pa12 strain by electroporation, and phage rebooting was confirmed via a plaque assay. White arrow heads indicate rebooted ΦR26 plaques produced on the lawn of the Pa12 strain.
53. Check the electrophoresis image and perform gel extraction and purification only if nonspecific bands are observed.

***Note:*** Subsequent experiments can be performed even if nonspecific bands are present. However, it may affect the efficiency of the desired assembly during seamless cloning. Conversely, gel extraction may reduce the sample yield.

52. Irradiate the stained DNA with UV light and check the image (Figure 2A).

For gel extraction (if required).

54. Mix Novel Juice 6× loading buffer (GeneDireX) with the remaining 40 μL of the DNA fragment sample not soaked in EtBr for gel extraction.
55. Mix 1 g of SeaKem ME Agarose (Lonza, Basel, Switzerland) with TAE buffer. Heat the solution to completely dissolve ME agarose in TAE buffer.
56. After mixing the Seakem ME Agarose (Lonza), prepare the gels for electrophoresis using a gel maker (Mupid, Tokyo, Japan) so that the thickness is 3–5 mm.
57. Pour 1× TAE buffer into the electrophoresis tank so that the top of the gel is soaked approximately 1 mm.
58. Apply the DNA fragment sample mixed with Novel Juice to the electrophoresis wells.
59. Set the electrophoresis conditions to 100 V for 35 min.
60. Cut out the desired bands using a scalpel under a BLooK LED Transilluminator.

***Note:*** To prevent DNA damage, we recommend using fluorescence-based imaging during gel extraction.

61. Extract DNA from the gel according to the protocol of the QIAquick Gel Extraction Kit (Qiagen, Hilden, Germany).
62. Purify all DNA fragment samples according to the protocol of the QIAquick PCR Purification Kit (Qiagen).
63. Elute the purified DNA fragments in 100 μL of nuclease-free water.
64. Add 3.3 μL of 3 M sodium acetate and 2 μL of etachinmate (Nippon Gene) to each reaction solution.
65. Mix the sample by vortexing.
66. Add 250 μL of 100% EtOH and mix thoroughly by vortexing.
67. Centrifuge the sample at 12,000 *× g* for 5 min and discard the supernatant.

***Note:*** If the desired concentration cannot be achieved from a single gel extraction, perform multiple extractions and concentrate the DNA using ethanol precipitation. To maximize assembly efficiency, ensure that the molar variation between fragments is within ±0.005 pmol.

***Note:*** At this time point, do not aspirate the precipitated white pellet DNA by mistake.

68. Add 1 mL of 70% EtOH and pipette gently to peel off the DNA precipitate adhering to the wall surface.
69. Centrifuge the sample at 12,000 *× g* for 2 min and discard the supernatant.
70. Turn the tube upside down and air-dry for 15 min.
71. When sufficiently evaporated, dissolve the DNA in 30 μL of nuclease-free water.
72. Measure the DNA concentration using Biospec-Nano (Shimadzu).
73. Dilute the DNA with nuclease-free water to a concentration of 0.05 pmol/fragment.

***Note:*** The DNA purification step in this section serves to eliminate the template phage DNA used during PCR, thereby preventing unintended rebooting of the original template phage. Our validation confirmed that the sequential purification procedures effectively reduced residual template DNA to undetectable levels.

### Seamless cloning and purification of the assembled product

**Timing: 1–2 days**

We successfully reconstructed the complete genome (65 kbp) of *Pseudomonas* virus ΦR26 from the 10 purified PCR-amplified DNA fragments. In this section, we describe an efficient seamless cloning method for assembling DNA fragments and detecting cloned DNA segments. Using this approach, the complete genomes of other phages can also be reconstructed. The long DNA molecules generated in this process must be highly accurate and yield high efficiency. Therefore, this study describes a method for the precise cloning of DNA fragments and obtaining a complete phage genome with high yield.

74. Mix the DNA fragment sample prepared in the “cloning and purification of phage DNA fragments” section in the following volume (for 10 fragments):

**Table:** DNA assembly reaction system

Reagent	Final concentration	Amount
DNA fragment	0.05 pmol	1 μL/fragment
NEBuilder HiFi DNA Assembly Master Mix	1×	10 μL
Nuclease-free water	N/A	Mix up to 10 μL if the DNA fragments are <10 μL
Total	N/A	20 μL

**CRITICAL:** The DNA fragments used for the seamless cloning of 10 fragments should be adjusted to high purity to improve the efficiency of assembly. Ensure to check for the absence of protein or ethanol contamination using a spectrophotometer or similar instrument. The recommended purity is OD260/280 = 1.75–1.9 and OD260/230 ≥2. If these conditions are not met, it is recommended to repeat the column purification in Step 62.

**CRITICAL:** The DNA fragments used for the seamless cloning of 10 fragments can be adjusted to equimolar concentration to improve the yield of the desired fragments. Ideally, the molar concentration of each fragment should be adjusted to within ±0.005 pmol using a spectrophotometer.

75. Incubate the sample mixture in a thermal cycler at 50°C for 2 h.

***Note:*** Although the general protocol requires 1 h at 50°C for the assembly of DNA fragments, we recommend extending the reaction time to 2 h to increase the yield.

76. Mix agarose S to 0.8% in TAE buffer. The solution is heated to completely dissolve the ME agarose in 1× TAE buffer.
77. After mixing agarose S, prepare the gels for electrophoresis using a gel maker (Mupid) so that the thickness is 3–5 mm.
78. Pour 1× TAE buffer into the electrophoresis bath so that the top of the gel is soaked approximately 1 mm.
79. Mix 1 μL of 6× loading buffer and 5 μL of the assembly product.
80. Apply 1-kb DNA Ladder RTU (GeneDireX) and the assembled product stained with 6× loading buffer to the wells of the agarose gel.
81. Set electrophoresis conditions to 100 V for 45 min.
82. After electrophoresis, stain with EtBr for 15 min for imaging, and check the integrity of DNA fragment ligation (Figure 2B).
83. To purify the assembled phage DNA for rebooting, mix up 15 μL of the assembly product not used for imaging to 100 μL with nuclease-free water.
84. Add equal volumes of phenol:chloroform:isoamyl alcohol (PCI) (Nacalai Tesque, Kyoto, Japan).
85. Pipette gently with a micropipette.
86. Centrifuge at 18,000 *× g* for 5 min at room temperature, and discard the supernatant.

***Note:*** Be careful not to aspirate the lower layer.

87. Add an equal volume of chloroform:isoamyl alcohol (CIA) (Nacalai Tesque) to the collected supernatant.
88. Pipette gently with a micropipette.
89. Centrifuge at room temperature, 18,000 *× g* for 5 min, carefully remove the aqueous layer (upper layer), and transfer it to another tube.
90. Confirm the purity of the obtained DNA samples using the BioSpec-Nano (Shimadzu) by measuring the OD260/280 and OD260/230 values.
91. Accurately quantify the DNA concentration using the Quantus Fluorometer (Promega, Madison, WI) with the QuantiFluor dsDNA System (Promega), according to the manufacturer’s protocol.

**CRITICAL:** If proteins are not removed by PCI and CIA treatment, they may cause issues such as arcing during electroporation.

### Preparation of competent cells from *P. aeruginosa* clinical isolates

**Timing: 1 day**

This section describes a rapid and reproducible protocol for preparing competent cells from the *P. aeruginosa* clinical isolate Pa12, optimized for electroporation of linear DNA fragments.

92. Inoculate 5 mL of LB broth with 20 μL of Pa12 glycerol stock and incubate overnight at 37°C with shaking.
93. Mix with 100 μL of Pa12 incubated overnight with 9.9 mL of LB broth (100-fold dilution).
94. Incubate the sample at 37°C for 3–4 h with shaking until the OD_600_ reaches 0.6.
95. Centrifuge the sample at 10,000 *× g* for 5 min and remove the supernatant, except for the pellet, by aspiration.
96. Pipette the pellet slowly in sterile water and resuspend.
97. Refill 1 mL of the suspension into another container.
98. Centrifuge it at 10,000 *× g* for 5 min, remove the supernatant, except for the pellet, by aspiration, and resuspend it by pipetting slowly with 1 mL of 10% glycerol solution. Perform this process twice.

***Note:*** To obtain a competent cell with the appropriate resistance, ions must be separated from the sample into the supernatant. It is recommended to pipette approximately 100 times with a 1-mL pipette tip to separate the ions.

***Note:*** If the measured resistance is too high, adding a small amount of TE buffer (Tris-EDTA) can help reduce the resistance.

99. Centrifuge the sample at 10,000 *× g* for 5 min, remove the supernatant, except for the pellet, by aspiration, add 500 μL of 10% glycerol solution gently over the pellet, transfer 100 μL to a cuvette, and measure the resistance value.

***Note:*** We recommend checking the actual resistance values of the competent cells. The recommended resistance is <10 kΩ. If the sample resistance is too high or too low, the appropriate voltage may not be applied to the competent cell.

***Note:*** Sucrose-based preparation of competent *P. aeruginosa* cells has also been reported to yield high electroporation efficiency,^20^ and we also successfully used this method to reboot phages in Pa12. However, our protocol is specifically optimized for the delivery of linear DNA fragments, which are more prone to intracellular degradation. To ensure sufficient DNA uptake per cell, a moderate cell density is required. Therefore, competent cells obtained from overnight cultures with high biomass may be suboptimal for this application.

***Note:*** Because the freeze–thaw cycle reduces transformation efficiency, it is recommended that the prepared competent cells be divided into smaller aliquots.

Storage: Competent cells can be stored for approximately 6–12 months at −80°C with high electroporation efficiency.

### Gene transfer by electroporation

**Timing: 2 days**

In this section, we describe how to introduce a complete phage genome toward the competent cell obtained in the previous section.

100. Inoculate 5 mL of LB broth with 20 μL of host bacteria and incubate it overnight at 37°C with shaking.
101. Measure 100 μL of the overnight-incubated host bacteria, mix it with 9.9 mL of LB broth, and dilute the culture 100-fold.
102. Incubate the diluted culture at 37°C with shaking for 3–4 h until the OD_600_ reaches 0.6.
103. Retrieve the competent cells prepared in the previous section from the stock and thaw them on ice.
104. Gently invert the thawed competent cells to mix them.
105. Add 100 μL of the competent cells to a NEPA cuvette electrode set and measure the resistance.
106. Add 200 ng of the complete reconstructed phage genome to the competent cells in the cuvette.
107. Set up a negative control by adding the same amount of solvent for the reconstructed phage genome.
108. Measure the resistance and confirm that it falls within the range of <10 kΩ.

***Note:*** Check for a slight decrease in resistance after the inserted DNA has been mixed with the competent cell sample.

***Note:*** If the resistance is >10 kΩ, add 1 μL of TE buffer to the sample, check the resistance each time, and adjust it until the appropriate value is reached.

109. Incubate the entire cuvette in ice for 5 min.
110. Perform electroporation under the following conditions:

**Table:** Electroporation system

Voltage (V)	Pulse width (ms)	Pulse interval (ms)	Number of times
2,600	3.5	50	1

***Note:*** If necessary, a recovery culture of electroporated cells can be performed for approximately 3 h in SOC medium.

Mix 100 μL of the electroporated sample in the NEPA cuvette with 100 μL of the nonelectroporated Pa12 cells prepared in Step 102), and plate the mixture as described. In parallel, mix 100 μL of the same DNA sample with 100 μL of the competent Pa12 cells without electroporation as a negative control to check for possible phage contamination. No plaques should be observed in this control if the DNA preparation is free of phage particles.

**CRITICAL:** In this protocol, untreated bacterial cells are mixed with electroporated bacterial cells. The untreated bacteria are responsible for compensating for the dead bacteria in electroporation and forming a lawn that facilitates the identification of phage plaques.

111. Prepare two autoclaved LB agar plates.
112. Place the LB top agar in a 50°C heat block to prevent solidification.
113. In a 1.5-mL tube, combine the mixed sample prepared in Step 110), add 200 μL of the sample to 3 mL of LB top agar, and mix the sample thoroughly.
114. Before the LB top agar solidifies, quickly overlay it onto the LB agar plate, tilting the plate to ensure an even distribution. Allow the agar to set until it completely solidifies.
115. Once the agar has solidified (approximately 5 min), invert the plate and incubate it overnight at 37°C.
116. Inoculate 5 mL of LB broth with 20 μL of host bacteria and incubate it overnight at 37°C with shaking.
117. The following day, examine the plate for plaque formation.
118. If plaque formation is observed or the entire surface is lysed, it indicates that the phage has been successfully rebootted (Figure 2B).

***Note:*** If plaque is observed on the negative control, it is recommended to reconsider the entire rebooting process, as plaque formation may be due to phage contamination or possible prophage induction caused by electroporation stress.

### Cloning and rapid propagation of rebooted phages

**Timing: 3 days**

This section describes how to purify and use the phage successfully rebooted in the previous section.

119. If plaques are identified, prepare pipette tips for picking by cutting off the tip of the pipette tip and autoclaving it at 121°C for 20 min.
120. Collect the plaque with the pipette tip for picking with the entire surrounding Pa12 lawn, place it in a tube containing 1 mL of LB broth, and incubate it overnight with shaking.
121. Centrifuge the sample at 10,000 *× g* for 5 mins, and collect 800 μL of the coculture supernatant in a 1.5-mL tube.
122. Filter-sterilize 800 μL of the coculture supernatant with a sterile 0.45-μm syringe filter and collect the phage solution.
123. Repeat the same procedure three times to ensure that they are clonal isolates.
124. In order to amplify clonal isolates, rebooted phages will be propagate and purified as described in the “phage stock preparation” section.

## Expected outcomes

**Figure 3:**
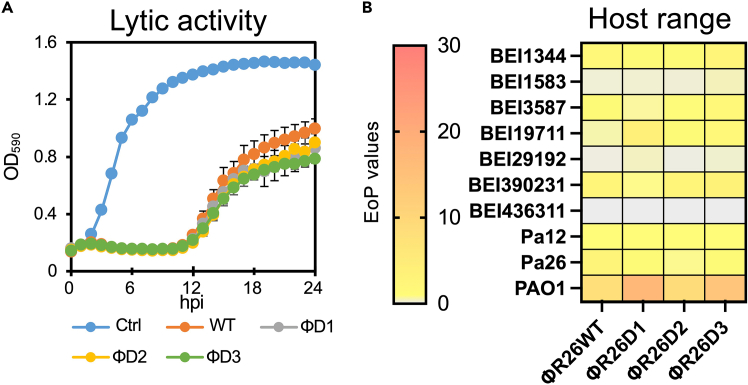
Validation of the lytic activity of rebooted ΦR26 strains (A) Lytic curves of parental ΦR26 and rebooted three ΦR26 strains, assigned as ΦD1, ΦD2, and ΦD3, respectively. Phages were inoculated into the Pa12 strain at an MOI of 0.1, and OD_590_ values were monitored every 1 h for 24 h at 37°C with shaking using a plate reader (Sunrise Rainbow Thermos RC, TECAN, Grödig Austria). The individual points in each lytic curve are presented as means ± SD (n = 3). (B) Phage host range determination performed using the EoP method. *P. aeruginosa* strains (Pa12 and Pa26 derived from animal infection sites,^3^^,^^18^^,^^19^ PAO1 purchased from ATCC, BEI strains derived from *P. aeruginosa* BEI panel^24^) were grown in LB medium overnight at 37°C with shaking. LB top agar containing *P. aeruginosa* strains was overlaid on an LB agar plate. Thereafter, 2 μL of diluted parental or rebooted ΦR26 aliquot (10^7^–10^1^ PFU/mL) in SM buffer was dropped onto an overlaid LB agar plate to observe the lytic activity of phages by plaque formation. EoP values represent PFU using the specific *P. aeruginosa* strain/PFU using Pa12.

This protocol is designed to enable rebooting *Pseudomonas* virus ΦR26 with DNA of 65 kbp. Several additional experiments should be performed to confirm that the parent and rebooted phages are exactly similar. Here is an example of exactly how to check if the phage rebooted by electroporation is completely identical to the parent phage. After whole-genome sequencing with 150-base paired-end reads of three rebooted clones of ΦR26 using DNBSEQ-G400 (MGI Tech, Shenzhen, China), using extracted phage DNA (as described in the “extraction of phage DNA” section), we evaluated the completeness of the phage genome compared with parental ΦR26. No single nucleotide variants, insertions, or deletions were detected (data not shown). The use of high-fidelity polymerase is critical to ensure sequence accuracy sufficient for reliable phage rebooting. Moreover, as depicted in Figure 3A, rebooted ΦR26 resulted in almost complete similar lytic activity against the propagating strain Pa12 compared with parental ΦR26 in the turbidity reduction assay as described elsewhere^19^ Furthermore, host range determination based on the efficacy of the plating (EoP) method^21^^,^^22^ showed that the killing spectrum of rebooted ΦR26 was completely similar to that of parental ΦR26 against representative 10 strains of *P. aeruginosa* (Figure 3B). The representative plaque-forming activity of parental ΦR26 and the rebooted phage (ΦR26D1) is illustrated in Figure S2. These examples demonstrate that our protocol can accurately reboot ΦR26 from its DNA reconstructed by the assembly of 10 PCR fragments *in vitro*. This protocol could be applied to generate phages with specific modifications by incorporating any desired genetic sequence during the fragment design stage in the future. Moreover, by examining the sequences of lysogenic phages in pathogenic bacteria and designing fragments to remove the repressor region,^23^ it is possible to convert lysogenic phages into virulent phages.

## Limitations

This protocol summarizes the workflow for rebooting *Pseudomonas* phages with genomes of approximately 65 kbp. Nevertheless, the conditions for each step must be adjusted depending on the host bacterium and phage strain that you will use. Phages and host strains are highly diverse, and their properties can vary significantly. Therefore, the experimental conditions should be flexible and adaptable. Another potential limitation of this protocol is the loss of DNA due to repeated purification steps aimed at improving the purity of DNA fragments and the viral genome simultaneously minimizing sample concentration errors as much as possible through close monitoring and essential optimization of DNA extraction. Typically, 2–3 plaques are detected on the lawn of the host *P. aeruginosa* strain per agar plate following electroporation. However, in some instances, no plaques may form. Therefore, preparing multiple plates for each rebooting experiment is recommended. Furthermore, plaque detection efficiency can be improved by using a host strain in which key phage defense systems, such as restriction-modification, CRISPR systems and CBASS, have been deleted or attenuated as described by Ipoutcha *et al.* for rebooting of *Pbunaviruses.*^8^

## Troubleshooting

### Problem 1

Insufficient amount of phage genomic DNA is acquired.

### Potential solution

• One of the simplest solutions is to strengthen the titer of the phage. Simply increasing the phage titer will improve the yield of phage genomic DNA.
• If you collect the eluate from the elution tube and pass it through the column again, you may be able to recover any residual DNA on the column.

### Problem 2

Electrophoresis image is unclear.

### Potential solution

• Check that the gel thickness is appropriate. If the gel is too thick, staining may brighten the background and make it difficult to observe the bands.
• If the DNA amount is too small, increase the concentration by ethanol precipitation to increase the concentration.
• Check to ensure that the electrophoresis bath is not excessively heated. Overheating of the electrophoresis bath may cause a temperature difference between the surface and center of the gel, which may result in unclear images.
• Check that the staining time with ethidium bromide is appropriate. If the staining time is too long, the background may become brighter, making it difficult to identify the bands. If the staining time is too short, the DNA emission may be weak, and it may be difficult to identify the band.

### Problem 3

Resistance of the competent cell is low or the addition of DNA causes a large decrease in resistance.

### Potential solution

• The major cause of low resistance of the competent cell is insufficient desalination. Gentle pipetting during competent cell preparation is important to sufficiently dissociate salts adhering to the cells into the solution and remove them as supernatant. If the resistance of the competent cell is low, increase the frequency of pipetting or ensure that the pellet is sufficiently resuspended in the solution.
• If the resistance decreases significantly with the addition of DNA, the DNA may not have been sufficiently purified. Ensure that impurities have been adequately removed through column purification or phenol/chloroform treatment. Furthermore, DNA purification using dialysis membranes, in addition to this protocol, may help maintain appropriate resistance values.

## Resource availability

### Lead contact

Further information and requests for resources and reagents should be directed to and will be fulfilled by the lead contact, Jumpei Fujiki (j-fujiki@rakuno.ac.jp).

### Technical contact

Technical inquiries regarding this protocol should be directed to and will be answered by the technical contact, Jumpei Fujiki (j-fujiki@rakuno.ac.jp).

### Materials availability

Materials associated with this protocol are available upon request from the lead contact.

### Data and code availability

This protocol does not report datasets or generate code.

## Acknowledgments

The authors are grateful to Dr. Tomohiro Nakamura (Azabu University, Japan, and University of California San Diego, US) for his suggestions and helpful advice. This study was supported by grants for Scientific Research on Innovative Areas and International Group from the MEXT/10.13039/501100001691JSPS KAKENHI (JP23K23791).

## Author contributions

Conceptualization, D.Y. and J.F.; data curation, D.Y., J.F., N.K., H.Y., and H.I.; formal analysis, D.Y. and J.F.; funding acquisition, J.F. and H.I.; investigation, D.Y., N.K., H.Y., and J.F.; methodology, Y.S. and J.F.; project administration, J.F.; resources, Y.S., J.F., and H.I.; software, J.F.; supervision, J.F. and H.I.; validation, N.K., H.Y., Y.S., J.F., and H.I.; visualization, D.Y. and J.F.; writing – original draft, D.Y. and J.F.; writing – review and editing, N.K., H.Y., Y.S., J.F., and H.I.

## Declaration of interests

The authors declare no competing interests.
