# Supplementary material for: Protocol for end-design-free rebooting of terminally redundant Pseudomonas phages in clinical isolates of Pseudomonas aeruginosa
Source: STAR Protoc. 2025 Aug 4;6(3):104012. doi: 10.1016/j.xpro.2025.104012 (PMC12345254; doi:10.1016/j.xpro.2025.104012)
Supplement: Document S1. Figures S1 and S2 [file mmc1.pdf]

Figure S1

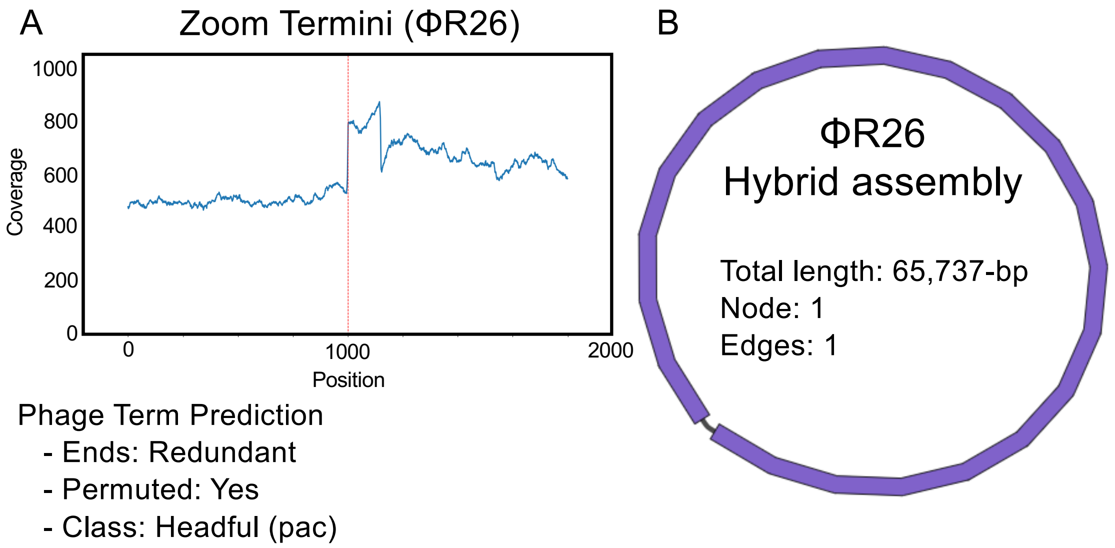

Figure S1. Characterization of the ΦR26 genomic architecture, related to Step 40 to 43 and “Before you begin”.
(A) To estimate the genome termini, a PhageTermVirome (4.3) analysis was performed using 150 bp paired-end short reads prepared with the NEBNext Ultra II DNA Library Prep Kit and sequenced on a NovaSeq X Plus (251 × coverage). The analysis revealed terminal redundancy and circular permutation of the genome, which is characteristic of a headful packaging mechanism. This indicates that the DNA molecule packaged within the ΦR26 virion is linear. (B) For complete genome assembly, DNA extracted from ΦR26 was sequenced using both NovaSeq X Plus (short reads) and MinION (long reads) platforms. A hybrid assembly of the data using Unicycler v0.4.8 yielded a single circularly permuted contig of 65,737 bp. The final assembly was supported by 243 × coverage from short reads and 12,108 × coverage from long reads.

Figure S2

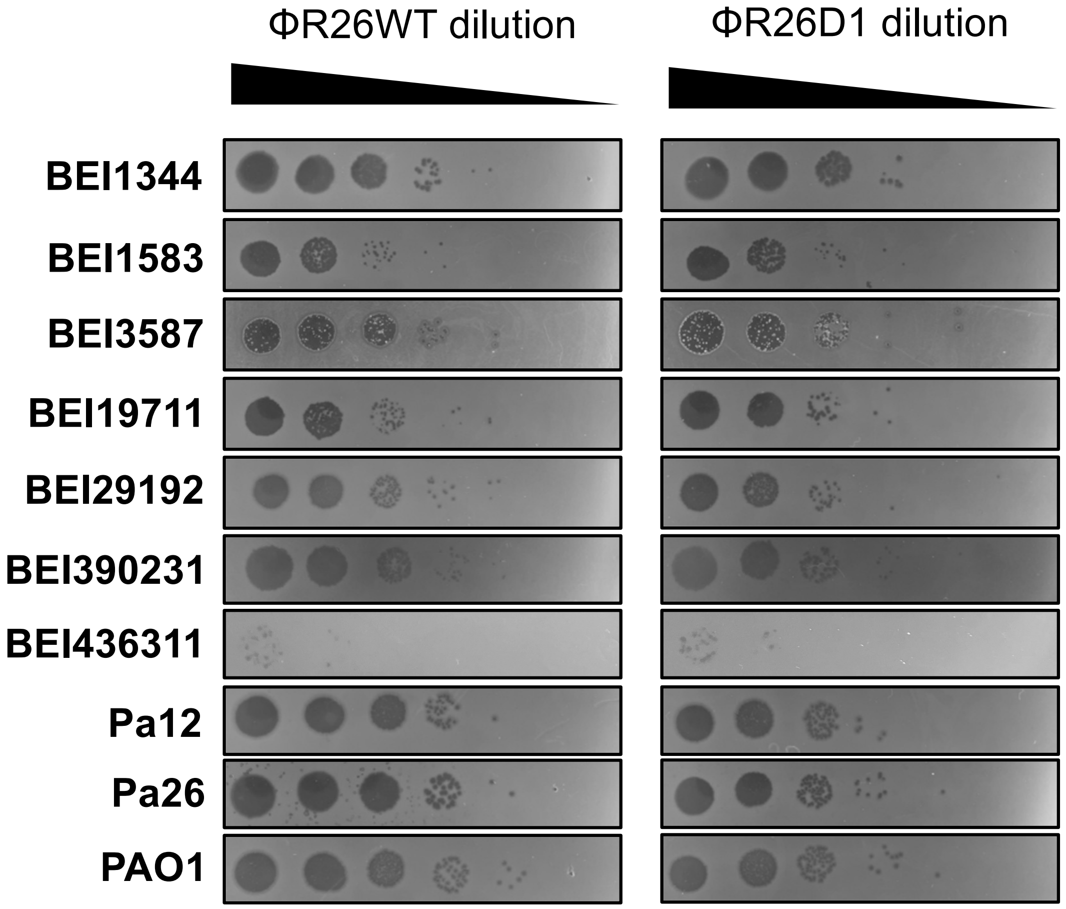

Figure S2. Plaque-forming Activity of Parental ΦR26 and Rebooted ΦR26, related to Step 124 and “Expected outcomes”

Representative plaque-forming activity of parental ΦR26 and rebooted ΦR26 (ΦR26D1). A 2-μL aliquot of phage dilutions (10^7–10^1 PFU/mL) was spotted onto the lawn of 10 strains of *P. aeruginosa*.
